# Autoimmune alopecia areata due to thymoma without myasthenia gravis: a case report

**DOI:** 10.1186/s40792-023-01655-2

**Published:** 2023-05-03

**Authors:** Yukino Saito, Tomohiro Yazawa, Toshiteru Nagashima, Yoichi Ohtaki, Natsuko Kawatani, Eiji Narusawa, Ryohei Yoshikawa, Nozomi Matsumura, Tatsuro Maehara, Ken Shirabe

**Affiliations:** 1 Division of General Thoracic Surgery, Integrative Center of General Surgery, Gunma University Hospital, 3-39-15, Showa-Machi, 371-8511 Maebashi, Gunma, Japan; 2 Department of Pathology, Gunma University Hospital, Gunma, Japan

**Keywords:** Thymoma, Alopecia areata, Surgery, Autoimmune disease

## Abstract

**Background:**

Thymoma is associated with autoimmune diseases. Myasthenia gravis is frequently associated with thymoma, but cases of thymoma complicated by alopecia areata are very rare. In this report, we present a case of thymoma associated with alopecia areata, but without Myasthenia gravis.

**Case presentation:**

A 60-year-old woman had a complaint of rapid progression of alopecia areata. A hair follicular biopsy was performed, which showed the infiltration of CD8-positive lymphocytes. She was prescribed topical steroids for 2 months prior to surgery, but her hair loss was not improved. Screening computed tomography showed a mass in the anterior mediastinum, which was suspected to be a thymoma. Myasthenia gravis was ruled out because she had no relevant symptoms or physical findings, and no anti-acetylcholine receptor antibodies were detected in serum. We performed a transsternal extended thymectomy based on a diagnosis of thymoma Masaoka stage I, without myasthenia gravis. Pathological examination showed Type AB thymoma, Masaoka stage II. The chest drainage tube was removed on postoperative day 1, and the patient was discharged on postoperative day 6. The patient has continued topical steroids and showed improvement 2 months postoperatively.

**Conclusions:**

Although alopecia areata is a rare complication in thymoma cases without myasthenia gravis, thoracic surgeons need to keep this condition in mind because alopecia reduces the patient's quality of life.

## Background

Thymoma is associated with autoimmune diseases. Myasthenia gravis (MG) is frequently associated with thymoma, but cases of thymoma complicated by alopecia areata are very rare [1]. When thymoma is associated with alopecia areata, it is characterized by the infiltration of CD8-positive lymphocytes in the hair follicles, and the majority of such cases are complicated with MG [2]. In this report, we present a case of thymoma associated with alopecia areata, but without MG.

## Case presentation

**Fig. 1:**
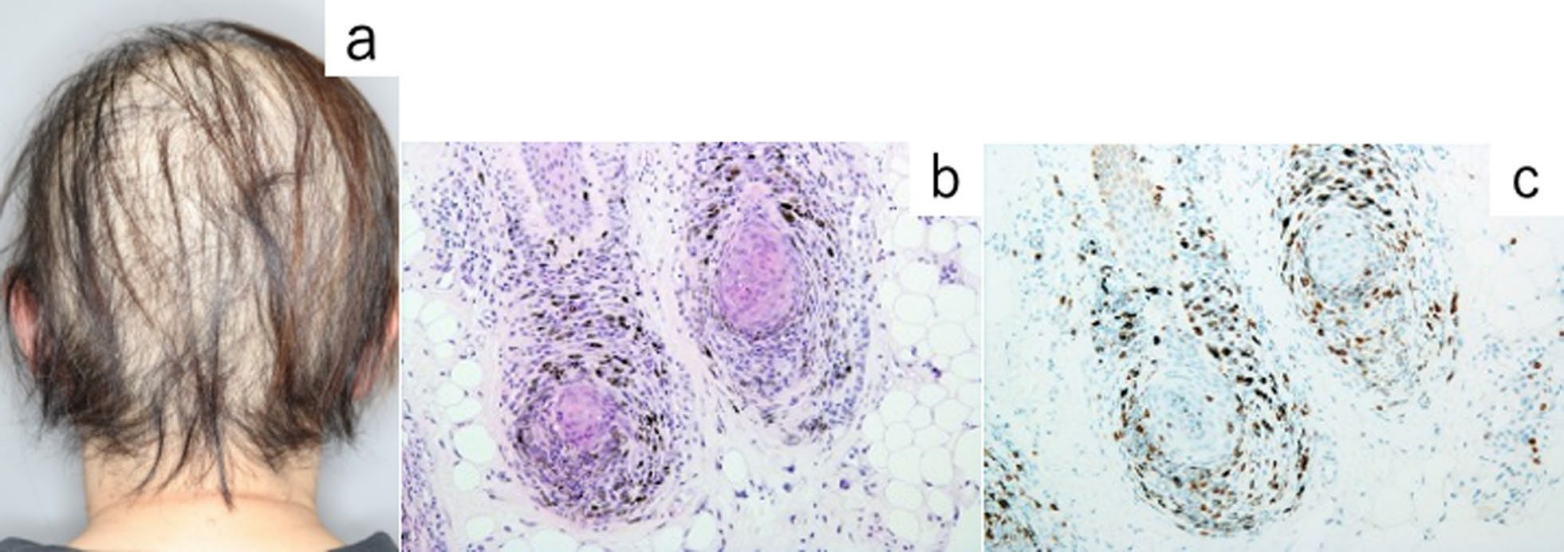
Physical examination and pathological findings of a follicular biopsy. **a** Alopecia areata on the head was observed to be progressing preoperatively, and hair loss was observed on the entire head. **b** Longitudinal section of the hair follicle (HE stained × 10). An inflammatory cellular infiltration of neutrophils, lymphocytes, and plasma cells can be seen from the hair follicle to the surrounding tissue. **c** Immunohistochemical staining was performed (CD8 immunohistochemical stained × 10). CD8-positive T lymphocytes were observed around the hair follicle

**Fig. 2:**
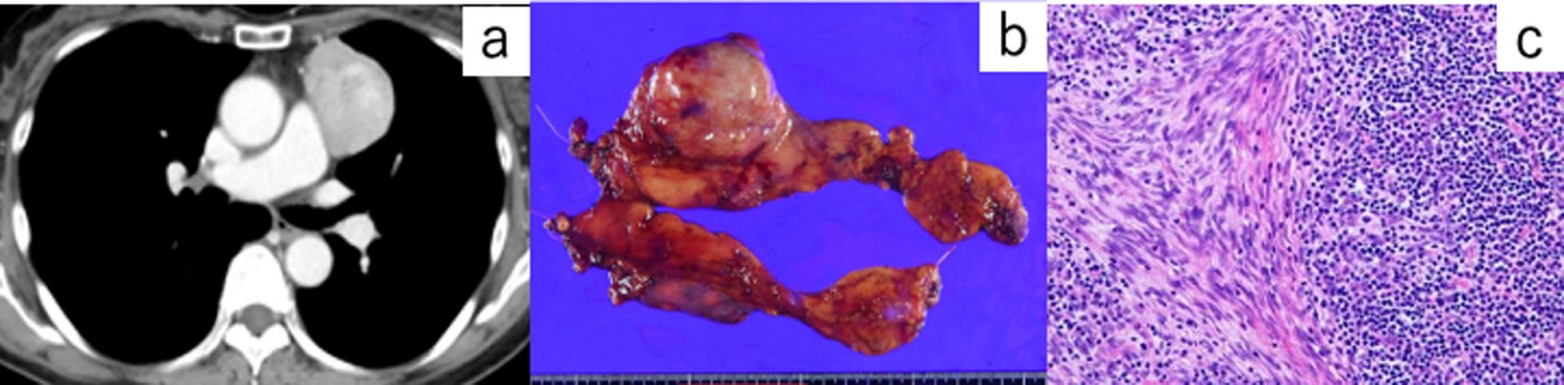
Thymoma. **a** Computed tomography image showing a well-defined mass of 6 cm on the left side of the anterior mediastinum. **b** Enlarged image of the excised thymus. The tumor was located on the left upper pole of the thymus. **c** Her thymoma showed type AB characteristics, which was a mixture of dark areas with abundant small atypical lymphocytes and areas of spindle-shaped cells with round to oval nuclei arranged in bundles

**Fig. 3:**
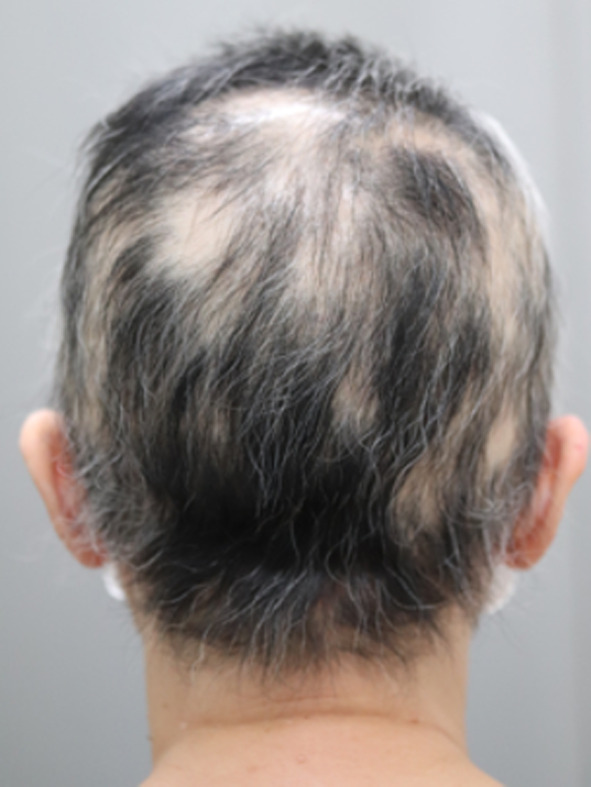
Physical examination at 6 month postoperatively. Alopecia areata is improving

A 60-year-old woman consulted her previous physician because of the rapid progression of alopecia areata (Fig. 1a). A follicular biopsy was performed, which showed the infiltration of CD8-positive lymphocytes; therefore, discoid lupus erythematosus was ruled out (Fig. 1b, c). She was prescribed topical steroids for two months prior to surgery, which were applied by the dermatologist to promote hair follicle regeneration, but her hair loss was not improved. Screening computed tomography (CT) showed a mass in the anterior mediastinum. She was then referred to our department. She had a medical history of hypertension, but did not have a smoking history. Chest CT showed a well-defined mass with a diameter of 6 cm on the left side of the anterior mediastinum, with no obvious evidence of infiltration of the surrounding organs (Fig. 2a). ^18F^Fluorodeoxyglucose positron emission tomography-CT showed a maximum standardized uptake value of 2.5, which was consistent with an anterior mediastinal mass. She had no symptoms and physical findings of MG, and laboratory testing revealed that no anti-acetylcholine receptor antibodies were detected in serum. Based on the above, she was diagnosed with thymoma Masaoka stage I, without MG. Transsternal extended thymectomy was performed (Fig. 2b); the operation time was 150 min, and the blood loss was 106 mL. Pathological examination showed type AB thymoma, Masaoka stage II (Fig. 2c). The chest drainage tube was removed on postoperative day 1, and she was discharged on postoperative day 6. She has continued topical steroids after surgery. Her hair loss was gradually improved from two months postoperatively, and further improvement in her hair loss has been observed without recurrence at 6 months postoperatively (Fig. 3).

## Discussion

Thymoma is often associated with autoimmune diseases in about one-third of cases [3], although the cases of thymoma associated with alopecia areata are rare. There are reports of alopecia areata in MG-associated thymoma, with a reported incidence of approximately 12%, and the condition does not depend on the degree of MG activity [2]. As MG is a common complication of thymoma, in the current case, a close examination for MG was also performed before surgery. Although MG was ruled out based on the blood test and clinical findings, we performed transsternal extended thymectomy because of the above report and the fact that MG often develops after thymoma surgery [4].

Thymoma complicated with alopecia areata but without MG is very rare. Alopecia areata is an organ-specific autoimmune disease caused by autoimmune T cells against hair follicle tissue [5]. Alopecia areata is characterized by the presence of CD8-positive lymphocyte infiltration in the hair follicle and decreases the patient’s quality of life. Suzuki et al. described alopecia areata as one of the non-motor symptoms of MG-associated thymoma and stated that it may be overlooked for the following reasons [2]; (1) thymoma-associated MG is not a common disease, and few patients are usually followed at a single institution; (2) MG is severe, and other symptoms are unnoted; (3) the neurologist, thoracic surgeon, and dermatologist follow up the patient separately; and (4) in addition to thymectomy, the patient may receive radiotherapy, chemotherapy, or immunotherapy, and thymoma-associated MG may be considered the side effect of these therapies.

Although alopecia areata could improve after thymoma resection, steroid therapy is often used [6]. Yu-Ri et al. reported a case of alopecia areata associated with thymoma without MG, which was improved in three months after surgical removal of a thymoma without steroid therapy [7]. To our best knowledge, this was the second case reported of alopecia areata associated with thymoma without MG. It is impossible to compare whether there is a difference in hair growth after thymectomy between thymoma complicated with MG and without MG, because there are very few reports of alopecia areata associated with thymoma without MG, so we are waiting for more such cases to be accumulated.

In addition, this case has not developed MG in the past or present. We would know when such a case would develop MG, however, to our best knowledge, this is only the second case reported, so we do not know at this time when a case of alopecia areata associated with thymoma without MG complication will develop MG. Further accumulation of cases will be necessary in the future.

## Conclusion

We thoracic surgeons need to note that alopecia areata may occur in thymoma cases. When such cases are encountered, not only is tumor resection necessary, multidisciplinary care in collaboration with neurologists and dermatologists is required to improve the patient's quality of life.

## Data Availability

Not applicable.

## Abbreviations

MG: Myasthenia gravis
CT: Computed tomography
